# Chemometric Assessment and Best-Fit Function Modelling of the Toxic Potential of Selected Food Packaging Extracts

**DOI:** 10.3390/molecules23113028

**Published:** 2018-11-20

**Authors:** Błażej Kudłak, Natalia Szczepańska, Miroslava Nedyalkova, Vasil Simeonov, Jacek Namieśnik

**Affiliations:** 1 Department of Analytical Chemistry, Faculty of Chemistry, Gdańsk University of Technology, 11/12 Narutowicza, 80-233 Gdańsk, Poland; nataliaszczepanska@vp.pl (N.S.); chemanal@pg.edu.pl (J.N.); 2 Chair of Inorganic Chemistry, Faculty of Chemistry and Pharmacy, University of Sofia “St. Kl. Okhridski”, 1, J. Bourchier Blvd., 1164 Sofia, Bulgaria; mici345@yahoo.com; 3 Chair of Analytical Chemistry, Faculty of Chemistry and Pharmacy, University of Sofia “St. Kl. Okhridski”, 1, J. Bourchier Blvd., 1164 Sofia, Bulgaria; vsimeonov@chem.uni-sofia.bg

**Keywords:** food packaging, canned food, ecotoxicity, assessment of extracts, chemometrics, best-fit function

## Abstract

Food packaging materials constitute an ever more threatening environmental pollutant. This study examined options to specifically assess the ecotoxicity of packaged wastes, such as cans, subjected to various experimental treatments (in terms of extraction media, time of exposure, and temperature) that imitate several basic conditions of the process of food production. The extracts were studied for their ecotoxicity with bioluminescent *Vibrio fischeri* bacteria. The first objective of this study was to find patterns of similarity between different experimental conditions; we used multivariate statistical methods, such as hierarchical cluster analysis, to interpret the impact of experimental conditions on the ecotoxicity signals of the package extracts. Our second objective was to apply best-fit function modelling for additional data interpretation, taking into account, that ecotoxicity for various temperature conditions is time- and temperature dependent. We mathematically confirmed that chemometric data treatment allows for better understanding how different experimental conditions imitating the real use of food packaging. We also demonstrate that the level of ecotoxicity depends on different extraction media, time of exposure, and temperature regime.

## 1. Introduction

Food packaging is indispensable for storing food at different temperatures. It extends the shelf life of the products and ensures protection from damage, influence of external factors, microbiological contamination, and loss of freshness and quality [1]. In highly developed countries, almost 95% of widely used products, especially food products, are sold in various types of packaging, which is made of plastics, paper, glass, metal, and composite materials [2].

Metal cans belong to the most frequently used Food Contact Materials (FCM) for both consumers and producers because of the intrinsic properties of metals. They are easy to form and are resistant to high temperatures and elevated pressure applied during the process of sterilization. Metal cans protect products from light and humidity and are lighter and more resistant to damage than glass, making the transport of such materials easier [3]. Despite these many advantages, metal cans expose the consumer to contact with various migrants. Numerous studies indicate that small molecules from package ingredients may migrate into food because of contact with the internal layer of the package and the technological processes applied during food processing [4,5,6]. With respect to metal packaging, xenobiotics originate mainly from the types of special coating used to protect the internal layer of the package against corrosion and to prevent direct contact between the food and the material from which the package was made [7]. The most common coatings are polyester coatings, acrylics, epoxy resins and vinylic organosols (also called polyvinyl chlorides). In the case of epoxy resins, layers are synthesized from bisphenol A (BPA) and its diglycidyl ether (BADGE). BADGE and its derivatives, formed as a result of contact with food, are most frequently identified in food samples [8,9]. The Commission Regulation (EU) No. 10/2011 on plastic materials and articles intended to come into contact with food contains permitted migration limits for compounds used in food packaging; the limits constitute a safe dose that does not cause risk to consumer health. According to the regulation, the sum of BADGE·HCl, BADGE·2HCl, and BADGE·HCl·H_2_O must not exceed 1 mg/kg in food or food simulant, and the sum of BADGE and its hydrolysed derivatives must not exceed 9 mg/kg [10,11]. The analysis of existing data shows that in the majority of cases, these compounds are present in food samples at low or very low levels of concentration; indeed, the levels of these compounds (BADGE and its hydrolysed derivatives [8,9]) are substantially lower than threshold values set forth in the Regulation. There is a lack of consensus in the scientific community on whether the migration limits set in the Regulation reflect the safety of the dose approved for human consumption, and whether exposing consumers to negligible amounts of contamination really does not constitute a risk to their health. These questions mainly concern a large group of contaminants known as Endocrine Disrupting Compounds (EDCs), which reach food and the human organism. EDCs contribute to a wide range of diseases and disabilities, including obesity, diabetes, cancer, heart disease, reproductive health problems, as well as neurodevelopmental and neurodegenerative disorders [1,2]. The results of *in vitro* studies suggest that BADGE shows estrogenic and anti-androgenic activity [12]. Furthermore, even at nanomolar levels, BADGE can induce adipogenic differentiation in both stromal stem cells and preadipocytes [1]. Current knowledge about xenobiotics indicates that the presence of several compounds, even at minimal levels of concentration, may cause a toxic effect [13]. Therefore, is seems appropriate to apply diagnostic tools, using a living organism as an active element, to evaluate the real impact of small-molecule compounds released from an internal layer of a package. Despite its importance, the problem of utilizing biological methods in assessing quality of FCMs is not so common; as given in [14] studies with biotests on packaging are much less frequently performed than those with instrumental methods. The endocrine potential of FCM extracts is most often tested with yeast androgen/estrogen screens (YES/YAS) and Chemical Activated LUciferase gene eXpression (CALUX) assays; the cyto- and genotoxicity of paper and paperboard packaging was confirmed with DNA damage studies.

Chromatographic methods are frequently used to determine the impact of packaging on the quality of food. However, there is little information on the use of bioanalytical methods for evaluating the cumulative impact of leachable package ingredients on food samples. Combining ecotoxicological studies and chemometrics will provide valuable information on how packaging influences food quality and can evaluate the risk of consuming food stored in metal packaging (with polymer lining constituting specific barrier between food and metal itself).

## 2. Results

### 2.1. Exploratory Data Analysis

**Figure 1:**
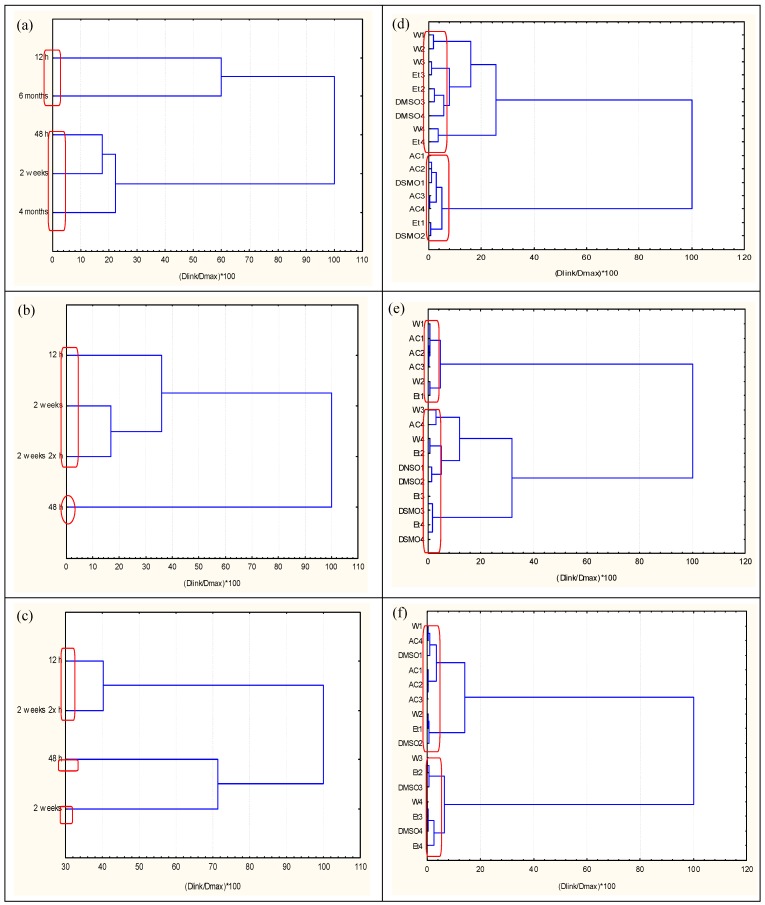
Hierarchical dendrogram for clustering of: (**a**) variable–time of extraction (RT data), (**b**) variable–time of extraction (65 °C), (**c**) variable–time of stay (121 °C data), (**d**) objects–extraction media (RT data), (**e**) objects–extraction media (65 °C data), (**f**) objects–extraction media (121 °C).

The input data set was divided into three subsets: 25 °C, 65 °C, and 121 °C. Each subset consisted of 16 objects, indicating packaging treated with: water, 3% acetic acid, 5% ethanol, and 5% DMSO (blanks were always used for all solvents and simulation media used as reference toxicity signal value). The codes W1–W4 indicate water, AC1–AC4 acetic acid, Et1–Et4 ethanol, and DMSO1–DMSO4 dimethylsulfoxide. The numbers 1 to 4 indicate the effective concentration of the extraction medium starting with 89.10% (high extractant concentration) to 10.24% (low extractant concentration) with dilution factor of 2. Times of treatment were designated as the variables, with 5 periods for room temperature (12 h, 48 h, 2 weeks, 4 months, and 6 months) and 4 periods for the other two temperatures (12 h, 48 h, 2 weeks, and 2 weeks × 2); the “× 2” notation indicates that the given sample was heated twice to stated temperature. Hierarchical dendrograms in Figure 1a–f show linkage between variables and objects using z-transform of input data, squared Euclidean distances as measure of similarity, Ward’s method of linkage, and Sneath’s criterion for cluster significance [15,16,17].

### 2.2. Best-Fit Function Modelling

Next, we investigated whether experimental input data can be subjected to best-fit function modelling in order to adequately describe the relationship between ecotoxicity (dependent variable) and concentration of the extraction medium (independent variable) for different experimental conditions (time and temperature of exposure). Three different best-fit regression functions were tested (linear, logarithmic, and polynomial). The multiple correlation coefficient *r*^2^ was chosen as validity measure for calculated/experimentally observed outputs. Table 1 presents a comparison of *r*^2^-values for the different models. All the examined options delivered adequate models; however, the polynomial model (Equation (1)) was the best-fit model for all tested experimental conditions:

*Y* = a_1_·*X*^2^ + a_2_·*X* + b (1)

**Figure 2:**
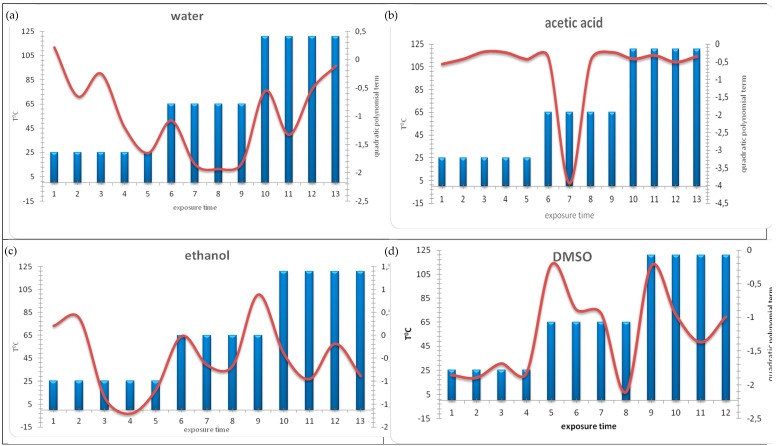
Change of impact of exposure time on ecotoxicity signal in (**a**) water samples for three different temperatures, (**b**) acetic acid medium for two different temperatures, (**c**) in ethanol medium for three different temperatures, (**d**) DMSO medium for three different temperatures.

Electronic Supplementary Table S1 presents the models obtained for each extraction medium, times of exposure, and temperatures. Table 1 presents the regression coefficients of the quadratic term for the respective polynomial models, which are used for the plots interpreted below (Figure 2). The confidence intervals for the correlation coefficients of the polynomial best-fit models for all extraction media at different times of treatment are presented in Electronic Supplementary Figure S2.

In Figure 2, the regression coefficient of the quadratic term in the polynomial models was used to measure the impact of the respective time of exposure and temperature for each extraction medium. The higher the value of the regression coefficient, the stronger the impact of the independent variable on the ecotoxicity signal. Nearly all the coefficients are negative, which has to be taken into account in the interpretation. The spline functions, used for the graphical presentation, do not indicate a specific relationship between the separate blocks of experimental conditions; 1–5 signify exposure times for 25 °C; 6–9 signify exposure times for 65 °C; 10–13 signify exposure times for 121 °C. The spline functions indicate only the dynamics of changes in the quadratic regression coefficient as relative measure for the impact of extraction media on the ecotoxicity.

## 3. Discussion

### 3.1. Discussion of Clustering Analyses

As indicated in the dendrograms, linkage of time exposure parameters (variables) for all temperatures was similar; short exposure times were linked to long periods of extraction (e.g., 12 h and 6 months for 25 °C; 12 h, 2 weeks, and 2 weeks doubly heated to 65 °C; 12 h and 2 weeks doubly heated to 121 °C). Conversely, intermediate time periods formed another cluster: 48 h, 2 weeks, 4 months for 25 °C; 48 h for 65 °C; 48 h and 2 weeks for 121 °C. These results indicate that changes in ecotoxicity responses are typical in the beginning of the extraction process, showing another level of toxicity during the intermediate period. Two major clusters of objects, with respect to the extraction media, were formed for all three temperature levels.

Conditionally, these two groups can be designated as “low toxicity” (LT) and “high toxicity” (HT) clusters:

(a) Room temperature (25 °C):

K1 (W1, W2, W3, W4, Et2, Et3, Et4, DMSO3, DMSO4)—*low toxicity*
K2 (AC1, AC2, AC3, AC4, Et1, DMSO1, DMSO2)—*high toxicity*

**Figure 3:**
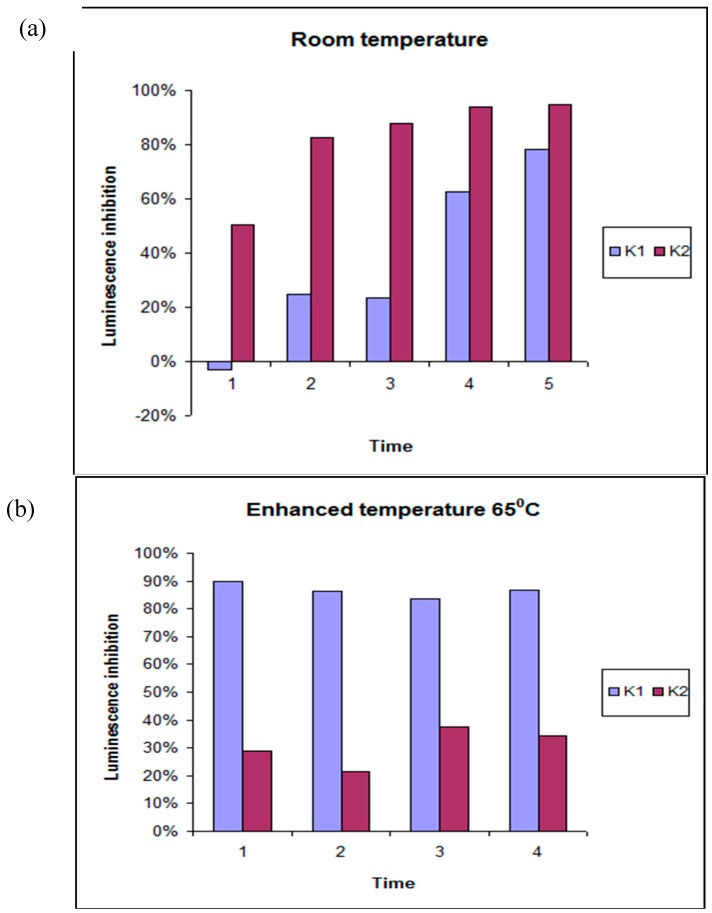

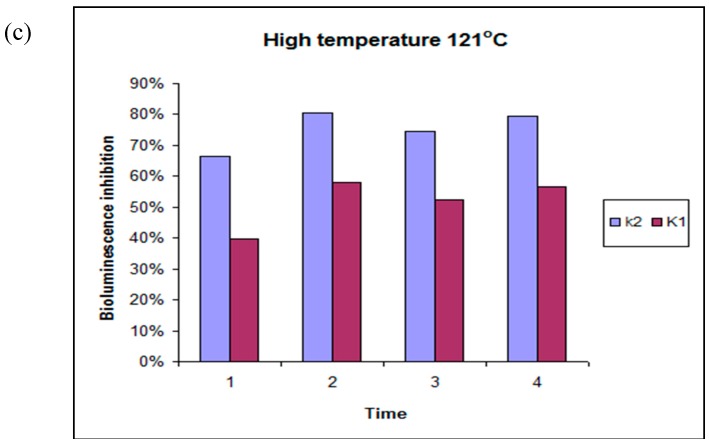
Luminescence inhibition (average values for clusters K1 and K2) as function of time at (**a**) room temperature, (**b**) enhanced temperature of 65 °C, (**c**) enhanced temperature of 121 °C (relative standard deviation of the means 3%).

The definition of “high” or “low” ecotoxicity is derived from the average values for inhibition of luminescence, which served as measure of ecotoxicity, for each cluster at any given time of exposure. The plot of ecotoxicological potential of clusters for extracts obtained at 25 °C is presented in Figure 3a.

The impact of time with respect to the extraction media (K1) can be clearly observed for water, ethanol, and low concentrations of DMSO. The same trend holds true for acetic acid, the highest concentration of ethanol, and high concentration of DMSO (K2), indicating that toxicity increases linearly with time. These results indicate that two patterns of toxicity formed for the RT extracts used in this study: low toxicity was related to water, ethanol, and low concentration of DMSO, while higher toxicity was linked to acetic acid and high concentrations of ethanol and DMSO. Both patterns indicate that the time of extraction considerably affected the level of toxicity.

(b) Temperature 65 °C:

K2 (W3, W4, AC4, Et2, Et3, Et4, DMSO1, DMSO2, DMSO3, DMSO4)—*low toxicity*
K1 (W1, W2, AC1, AC2, AC3, Et1)—*high toxicity*

Figure 3b shows the impact of time for both clusters. Increases in temperature influence both time and the extraction media. Ecotoxicological results of the K1 cluster (which includes acetic acid, high concentration of ethanol, and higher concentrations of the aqueous extracts of primary extracts samples) did not show time dependence. The same holds mostly true for the K2 cluster, which is correlated significantly with DMSO and ethanol extracts. No impact of time was apparent in the K2 cluster, which indicated only minimal toxicity, observed with the low toxicity pattern of the 48-h time period. A change in the level of toxicity with respect to the extraction media was observed at 25 °C; increased toxicity was associated with acetic acid and high concentrations of DMSO. However, at 65 °C, DMSO was associated with a lower toxicity level.

(c) Temperature 121 °C:

K1 (W3, W4, Et2, Et3, Et3, DMSO3, DMSO4)—*low toxicity*
K2 (W1, W2, AC1, AC2, AC3, AC4, Et1, DMSO1, DMSO2)—*high toxicity*

In general, at 121 °C, the impact of time was similar to that observed at 65 °C (Figure 3c); however, after 48 h of exposure, the pattern indicated lower toxicity, but not the lowest as observed at 65 °C. No significant time trend could be found at 121 °C. Table 2 presents a comparison for the toxicity levels of the different extraction media at different temperatures with respect to the two patterns identified by cluster analysis.

At 25 °C, water, ethanol, and extracts with lower concentrations of DMSO, are associated with reduced toxicity levels, while acetic acid and extracts with higher concentrations of DMSO are associated with elevated toxicity levels. For water-based extracts with a higher concentration of primary extract, preparation at elevated temperature resulted in increased toxicity; the toxicity levels of acetic acid- and ethanol-based extracts remained unchanged. Hierarchical cluster analysis data interpretation makes it possible to better understand the impact of different experimental conditions on the ecotoxicity readings of the extracted samples of packaging.

### 3.2. Discussion on Results of Best-Fit Function Modelling

A decrease in ecotoxicity was observed for the shortest times of exposure, reflected by the high negative values of the regression coefficient. The temperature factor, indicated by the three series of bars, is related to a decrease in ecotoxicity at low temperature (25 °C), constant value at intermediate temperature (65 °C), and a significant increase at the highest temperature (121 °C). This indicates that when water was used as the extraction medium, both temperature and time of exposure are significant factors affecting ecotoxicity.

In Figure 2b the results obtained by cluster analysis are confirmed; acetic acid as the extraction medium is characterized by high levels of ecotoxicity for various experimental conditions. The only exception is the minimal toxicity observed at 65 °C after 48 h of exposure. Therefore, for acetic acid, the influence of experimental parameters on ecotoxicity is not well expressed. No significant changes were observed with variation in time of exposure and temperature; the level of ecotoxicity remained high.

The plot showing ethanol used as extraction medium (see Figure 3c) indicates a trend similar to that of water, especially at 65 °C and 121 °C. Ecotoxicity decreased with time at RT and remained at a constant level at the 65 °C; the exception was an increase observed after a longer time of exposure. The difference with respect to water is the decrease in ecotoxicity observed at the highest temperature. Thus, we conclude that time and temperature are significant experimental factors when ethanol is used as the extraction medium. The results of chemometric interpretation are summarized in Supplementary Table S2.

As seen in Figure 2d the toxicological behavior of DMSO differs from those of the other extraction media. At low temperature, no change in ecotoxicity was observed with respect to time. For the other two temperatures, a similar pattern of change in ecotoxicity occurred; ecotoxicity increased after short times of exposure followed by a decrease after longer times. Again, time of exposure and temperature were significant factors affecting the level of ecotoxicity.

The physical meaning of the regression coefficient for the quadratic term in the model can be summarized as follows:

- positive coefficients—indicate minimal ecotoxicity and negative values of bioluminescence inhibition;
- low negative coefficient—indicates higher or intermediate ecotoxicity and low positive values of bioluminescence inhibition;
- very negative coefficient—indicates maximal ecotoxicity and high positive values of bioluminescence inhibition.

It should be kept in mind that the spline in Figure 2 does not represent a model of the dependence between ecotoxicity and the experimental conditions studied, but indicates only the dynamic variation of the ecotoxicity impact as assessed by the quadratic regression terms from the regression signal/experimental conditions. Therefore, no model validation for the spline “model” is needed. The major goal is to assess in a semi-qualitative way the relationships mentioned above which will be of practical use. This is a rather black box approach where the mechanism of transformation of the input factors to the output signal is unknown.

## 4. Materials and Methods

### 4.1. Instruments, Chemicals, and Reagents

The Microtox^®^ 500 kit (2% NaCl, lyophilized *Vibrio fischeri*, Microtox Diluent, Microtox Acute Reagent, Osmotic Adjusting Solution (OAS), and Reconstitution Solution (RS) was purchased from ModernWater Ltd. (Cambridge, GB). Ethanol (EtOH, CAS no. 64-17-5), dimethyl sulfoxide (DMSO, CAS no. 67-68-5), acetic acid (CAS no. 64-19-7), and Parafilm^®^ were purchased from Sigma-Aldrich (Steinheim, Germany). All reagents were of analytical grade or higher; reagents used for microbiological purposes. The instruments and equipment used during the study were: Microtox^®^ 500 from Modern Water Ltd., electronic multi- and single-channel pipettes from Eppendorf (Darmstadt, Germany), CP411 pH-meter from Metron (Zabrze, Poland), and shaker type water bath 357 from Elpan Laboratory Instruments (Lubawa, Poland). Metal cans used specifically for fish storage were obtained from a local producer in Ustka (Ustka, Poland).

### 4.2. Sample Preparation

The studies on identifying material of interior layer of the packaging are described in [18]. The total area of each container was c.a. 180 cm^2^. Three simulants reflecting specific kinds of food have been chosen with respect to the methodologies used in order to assess global migration, as described in Commission Regulation (EU) No. 10/2011 on plastic materials and articles intended to come into contact with food; distilled water for aqueous foods with a pH above 4.5; acetic acid at 3% in distilled water for acidic aqueous food with pH below 4.5; ethanol at 5% for any food that may contain alcohol. Examples of standard procedures in the EU legislation recommend using 10% EtOH; however, due to high alcohol content, there was unintended increase in toxicity, and lower ethanol content had to be used; DMSO is used to reflect behavior of simulants of lipophilic character. Cans covered with lids were sealed with Parafilm^®^ and shaken for 12 h, 48 h, 2 weeks, 4 months, and 6 months. All the time periods were subjected to different temperatures (at normal pressure): room temperature (RT) = 25 °C, 65 °C, and 121 °C, to reflect the processing conditions of food production with respect to guidelines stated in the Commission Regulation (EU) No. 10/2011 on plastic materials and articles intended to come into contact with food. Selected samples were also heated twice to study the impact of such processing on degradation of the internal resin layer lining the cans and release of contaminants into different extractants.

### 4.3. Toxicity Studies Using Microtox^®^

The acute toxicity of extracts was measured with Microtox^®^ biotest using marine bioluminescent bacteria *Vibrio fischeri* as the active element. The degree of toxicity was assessed based on inhibition of bioluminescence of the indicator organism. Appearance of factors that negatively influence the enzymatic activity of the indicator organism inhibits the oxidation of luciferin, which is manifested as reduced luminescence. Because these bioluminescent organisms are highly sensitive to the presence of toxic ingredients, they are used in environmental research to control the degree of toxicity in water, sewage, soils, sludge, ionic liquids, and nanoparticles, among others [15,19] For quality assurance, the following parameters were set according to manufacturer’s guidelines: for Microtox^®^ I_0_ of bacterial suspension >70 U, chromium sulphate was used as positive control of bacterial stock suspension.

The experimental design and toxicological studies are presented in our previous work [16]. Briefly, lyophilized reagent with *Vibrio fischeri* bacteria was hydrated with 1000 µL of RS and maintained at 5.5 ± 1.0 °C temperature. Then, 100 µL of the bacterial solution and samples of the standard dissolved in distilled water were added to the vials. To produce a suitable osmotic pressure above 2%, OAS was added to the vial with the highest concentration, and serial dilutions were prepared using the diluent. Incubation time for studies with bacteria was 30.0 min, according to the manufacturer’s protocol (in case of discoloring of samples also color adjustment procedure was applied).

### 4.4. Chemometric Data Treatment

Because the data set involves multifunctional conditions, such as extraction media, time, and temperature, we used chemometrics to interpret the experimental results. With traditional data mining options like hierarchical cluster analysis [17,20] it is possible to assess the impact of the various experimental conditions on the ecotoxic response of can packaging. Best-fit function modelling was performed to validate the conclusions obtained from multivariate statistics. The mathematical representative model is a precision tool for evaluating the behavior and dynamics of a system, enabling answers to questions about the different states of the system, and making respective predictions. In this work, we focused on analyzing models that can describe the input/output modes of each experimental state, such as polynomial regression models. Polynomials are often used when a simple empirical model is required. The polynomial model is used for interpolation or extrapolation, or to characterize data using a global fit. The main advantages of polynomial fits include reasonable flexibility for data that are not overly complex and linearity, which enables a simple fitting process. If there is no a priori information, general functions cover all different models depending on the regression coefficient. Modelling is necessary for interpreting states set forth by input conditions, especially time-dependent factors, because modelling reveals time-dependent changes in the states of a system.

Different models (linear, logarithmic, and quadratic polynomial) were tested to evaluate best-fit functions describing the experimental results. Regarding the correlation coefficient (such as experimental or calculated data), the polynomial model has been proven to adequately describe experimental data. Comparison between conclusions derived by data mining, and the regression coefficient for the quadratic term of the polynomial model for experimental conditions, provide an overview of the impact these conditions have on the ecotoxicity of the examined packaging.

Because cluster analysis is a well-documented and widely applied chemometric method, no detailed description of its theoretical background is presented in this manuscript. All calculations were performed using the STATISTICA 8.0 software package (StatSoft, Inc. (2007). STATISTICA (data analysis software system), version 8.0. www.statsoft.com. USA).

## 5. Conclusions

We analyzed the selected bioassay as tools for assessing the impact of metal cans as packaging with respect to time of contact with stored material, degradation at elevated temperatures, preservation/storage, and the type of product stored inside. Chemometric data interpretation made it possible to better understand the impact of different experimental conditions on the ecotoxicity readings of extracted packaging material. Packaging material was subjected to different treatment conditions to imitate real scenarios of food storage and eventual packaging wastes. Assessment of ecotoxicity of the packaging material was conducted by:

- hierarchical cluster analysis of input data,
- best-fit function modelling using the value of the regression coefficients from the derived polynomial models.

We showed that chemometric data interpretation allows for better understanding of different experimental conditions imitating the real use of food packaging. We also demonstrate that the level of ecotoxicity is dependent on different extraction media, time of exposure, and temperature regime.

It can be also concluded that combined biological-instrumental-chemometric studies constitute only a small fraction of overall packaging studies, and more efforts is needed to combine standardized analytical and bioanalytical solutions to study impact of real-scenario extraction conditions on degradation of FCM and its impact on food quality.

## Figures and Tables

**Table 1:** Correlation coefficients for the models tested.

WATER	***r*^2^**	12 h	48 h	2 Weeks	4 Months	6 Months	12 h	48 h	2 Weeks	2 Weeks 2x h	12 h	48 h	2 Weeks	2 Weeks 2x h
	linear	0.857	0.987	0.994	0.598	0.605	0.745	0.842	0.605	0.783	0.982	0.838	0.984	0.857
	log	**0.991**	0.970	0.878	0.855	0.855	0.939	0.960	0.853	0.964	0.952	0.986	0.956	0.990
	Polynom.	0.983	**0.995**	**0.995**	**0.956**	**0.928**	**0.940**	**0.992**	**0.918**	**0.995**	**0.996**	**0.992**	**0.998**	**0.997**
**ACETIC ACID**	***r*^2^**													
	linear	0.890	0.641	0.492	0.709	0.651	0.644	0.377	0.783	0.783	0.936	0.871	0.618	0.652
	log	0.960	0.880	0.766	0.927	0.873	0.880	0.643	0.963	0.964	0.998	0.975	0.849	0.891
	Polynom.	**0.985**	**0.934**	**0.897**	**0.981**	**0.888**	**0.991**	**0.775**	**0.966**	**0.976**	**0.990**	**0.980**	**0.870**	**0.950**
**ETHANOL**	***r*^2^**													
	linear	0.998	0.989	0.948	0.648	0.582	1.000	0.983	0.970	0.955	0.992	0.948	0.948	0.983
	log	0.890	0.847	0.973	0.871	0.835	0.916	0.970	0.985	0.993	0.956	0.994	0.994	0.945
	Polynom.	**1.000**	**0.998**	**0.995**	**0.886**	**0.906**	**1.000**	**1.000**	**0.998**	**0.995**	**1.000**	**0.991**	**0.991**	**0.986**
**DMSO**	***r*^2^**													
	linear	0.818	0.886	0.799	0.821	1.000	0.969	0.953	0.933	0.735	0.994	0.905	0.800	0.896
	log	0.978	0.998	0.963	0.977	1.000	0.980	0.968	0.982	0.920	0.951	0.990	0.971	0.996
	Polynom.	**0.994**	**0.994**	**1.000**	**0.999**	**1.000**	**0.990**	**0.993**	**0.997**	**0.996**	**1.000**	**1.000**	**0.975**	**0.999**

**Table 2:** Toxicity expertise for extraction media and temperature conditions.

EXTRACTION MEDIUM	RT	65 °C	121 °C
***W1***	LT	***HT***	***HT***
***W2***	LT	***HT***	***HT***
***W3***	LT	LT	LT
***W4***	LT	LT	LT
***AC1***	***HT***	***HT***	***HT***
***AC2***	***HT***	***HT***	***HT***
***AC3***	***HT***	***HT***	***HT***
***AC4***	***HT***	LT	***HT***
***Et1***	***HT***	***HT***	***HT***
***Et2***	LT	LT	LT
***Et3***	LT	LT	LT
***Et4***	LT	LT	LT
***DMSO1***	***HT***	LT	***HT***
***DMSO2***	***HT***	LT	***HT***
***DMSO3***	LT	LT	LT
***DMSO4***	LT	LT	LT

## Supplementary Materials

The following are available online, Figure S1: Analytical methodology of acute toxicity determination in the studies presented. Table S1: Summarized presentation of the quadratic regression coefficient for different experimental conditions and media, Table S2: Models obtained for each extraction medium, times of extraction and temperatures. Figure S2. Confidence intervals (95%) of *r*^2^ for the extraction media water (1), acetic acid (2), ethanol (3) and DMSO (4).
