# Supplementary material for: Chemometric Assessment and Best-Fit Function Modelling of the Toxic Potential of Selected Food Packaging Extracts
Source: Molecules. 2018 Nov 20;23(11):3028. doi: 10.3390/molecules23113028 (PMC6278273; doi:10.3390/molecules23113028)
Supplement: Supplementary file 1 [file molecules-23-03028-s001.pdf]

Electronic Supplementary Material

Article

Chemometric Assessment and Best-fit Function Modelling of the Toxic Potential of Selected Food Packaging Extracts

Błażej Kudłak [1,*], Natalia Szczepańska [1], Miroslava Nedyalkova [2], Vasil Simeonov [3] and Jacek Namieśnik [1]

[1] Department of Analytical Chemistry, Faculty of Chemistry, Gdańsk University of Technology, 11/12 Narutowicza, 80-233 Gdańsk, Poland; nataliaszczepanska@vp.pl (N.S.); chemanal@pg.edu.pl (J.N.)

[2] Chair of Inorganic Chemistry, Faculty of Chemistry and Pharmacy, University of Sofia "St. Kl. Okhridski", 1, J. Bourchier Blvd., 1164 Sofia, Bulgaria; mici345@yahoo.com

[3] Chair of Analytical Chemistry, Faculty of Chemistry and Pharmacy, University of Sofia "St. Kl. Okhridski", 1, J. Bourchier Blvd., 1164 Sofia, Bulgaria; vsimeonov@chem.uni-sofia.bg

* Correspondence: blakudla@pg.edu.pl; Tel.: +48-58-347-2128

Table S1. Summarized presentation of the quadratic regression coefficient for different experimental conditions and media*

Medium_T	12h	48h	2 weeks	Long term
W_RT	0.23	-0.65	-0.25	-1.20
W_65	-1.08	-1.08	-2.72	-1.84
W_121	-0.11	-1.33	-0.52	-0.11
AC_RT	-0.55	-0.42	-0.21	-0.23
AC_65	-0.36	-3.94	-0.46	-0.23
AC_121	-0.55	-0.42	-0.21	-0.23
Et_RT	0.21	0.38	-1.36	-1.70
Et_65	-0.02	-0.65	-0.67	-0.89
Et_121	-0.41	-0.96	-0.19	-0.89
DMSO_RT	-1.86	-1.90	-1.69	-1.83
DMSO_65	-0.21	-0.88	-0.94	-2.11
DMSO_121	-0.24	-0.96	-1.37	-0.99

* since temperature conditions the exposure time at room slightly differ from the time exposures for the other temperatures "long term" column presents models for period longer than 2 weeks

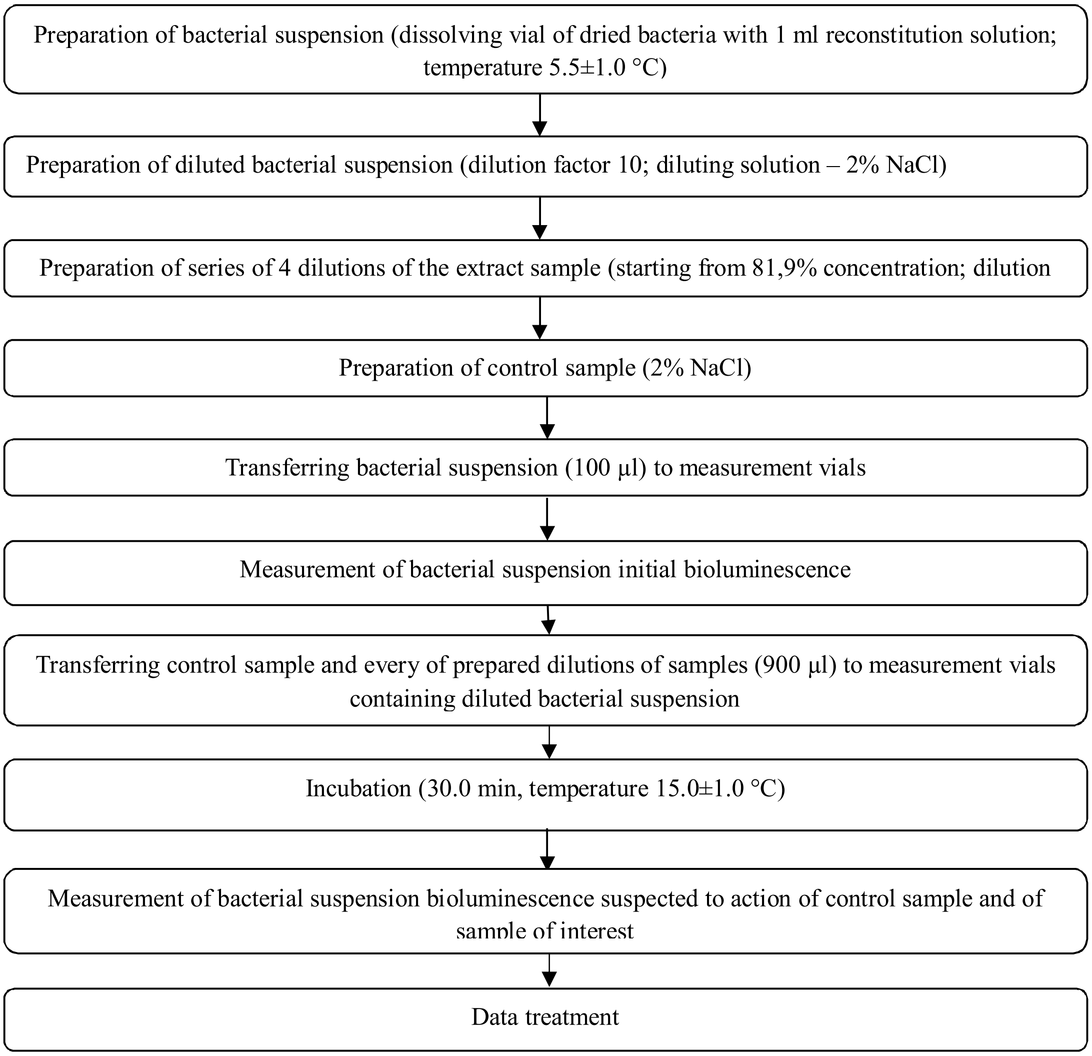

Figure S1. Analytical methodology of acute toxicity determination in the studies presented.

Table S2. Models obtained for each extraction medium, times of extraction and temperatures

Water/room temperature			Acetic acid/room temperature		Ethanol/room temperature		DMSO/room temperature	
Time	Polynomial model	R^2	Polynomial model	R^2	Polynomial model	R^2	Polynomial model	R^2
12h	$y = 0.228x^2 - 0.349x$	0.98	$y = -0.558x^2 + 0.913x + 0.226$	0.99	$y = 0.207x^2 + 0.684x - 0.226$	0.99	$y = -1.859x^2 + 2.705x - 0.273$	0.99
48h	$y = -0.651x^2 + 1.691x$	0.98	$y = -0.416x^2 + 0.549x + 0.839$	0.93	$y = 0.376x^2 + 0.390x - 0.106$	0.99	$y = -1.897x^2 + 3.028x - 0.342$	0.94
2 weeks	$y = -0.249x^2 + 0.912x$	0.97	$y = -0.208x^2 + 0.259x + 0.934$	0.9	$y = -1.361x^2 + 2.62x - 0.41$	0.99	$y = -1.686x^2 + 2.399x - 0.067$	1.00
4 months	$y = -1.203x^2 + 1.545x + 0.443$	0.96	$y = -0.233x^2 + 0.315x + 0.905$	0.98	$y = -1.705x^2 + 2.309x + 0.149$	0.89	$y = -1.830x^2 + 2.662x + 0.065$	0.99
6 months	$y = -1.624x^2 + 2.1103x + 0.269$	0.93	$y = -0.4236x^2 + 0.5746x + 0.8092$	0.89	$y = -1.181x^2 + 1.528x + 0.463$	0.91	No model possible	-

Water/65 ^{0}C			Water/121 ^{0}C		Acetic acid/65 ^{0}C		Acetic acid/121 ^{0}C	
Time	Polynomial model	R^2	Polynomial model	R^2	Polynomial model	R^2	Polynomial model	R^2
12h	$y = -1.082x^2 + 1.906x$	0.96	$y = -0.111x^2 + 0.997x$	0.98	$y = -0.356x^2 + 0.461x + 0.872$	0.99	$y = -0.356x^2 + 0.461x + 0.872$	0.99
48h	$y = -1.082x^2 + 1.906x$	0.96	$y = -1.331x^2 + 2.016x$	0.99	$y = -3.93x^2 + 4.80x - 0.1725$	0.78	$y = -0.314x^2 + 0.455x + 0.820$	0.98
2 weeks	$y = -2.721x^2 + 3.409x$	0.85	$y = -0.517x^2 + 1.369x$	0.99	$y = -0.460x^2 + 0.662x + 0.694$	0.97	$y = -0.502x^2 + 0.672x + 0.786$	0.87
2 weeks 2x	$y = -1.838x^2 + 2.616x$	0.99	$y = -0.111x^2 + 0.997x$	0.98	$y = -0.227x^2 + 0.324x + 0.891$	0.98	$y = -0.344x^2 + 0.454x + 0.857$	0.95
Ethanol/65 ^{0}C			Ethanol/121 ^{0}C		DMSO/65 ^{0}C		DMSO/121 ^{0}C	
Time	Polynomial model	R^2	Polynomial model	R^2	Polynomial model	R^2	Polynomial model	R^2
12h	$y = -0.017x^2 + 0.964x - 0.069$	1.000	$y = -0.408x^2 + 1.347x - 0.291$	0.99	$y = -0.215x^2 + 0.509x - 0.003$	0.99	$y = -0.237x^2 + 0.852x - 0.021$	1.00
48h	$y = -0.655x^2 + 1.659x - 0.208$	1.000	$y = -0.955x^2 + 1.868x - 0.193$	0.99	$y = -0.884x^2 + 1.766x - 0.183$	0.99	$y = -0.962x^2 + 1.578x + 0.18$	0.99
2 weeks	$y = -0.666x^2 + 1.459x - 0.107$	0.99	$y = -0.187x^2 + 0.888x - 0.167$	0.99	$y = -0.942x^2 + 1.682x - 0.098$	0.99	$y = -1.369x^2 + 1.989x - 0.011$	0.98
2 weeks 2x	$y = -0.891x^2 + 1.779x - 0.238$	0.99	$y = -0.889x^2 + 1.632x - 0.096$	0.99	$y = -2.105x^2 + 2.865x - 0.256$	0.99	$y = -0.992x^2 + 1.597x + 0.059$	0.99

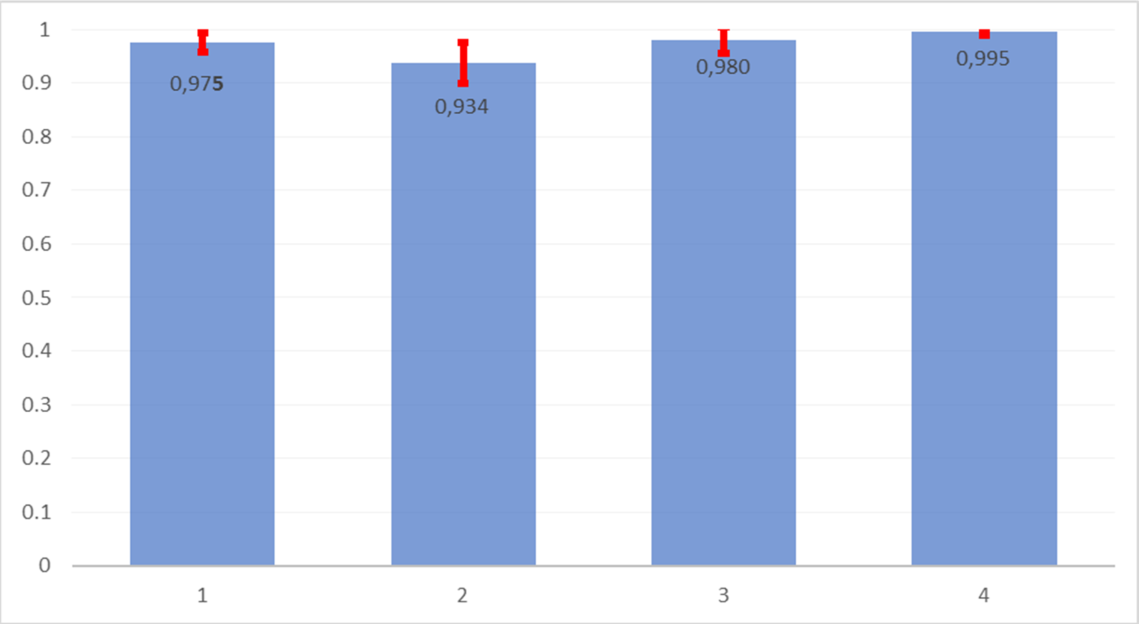

Figure S2. Confidence intervals (95%) of r^2 for the extraction media – water (1), acetic acid (2), ethanol (3) and DMSO (4).
